# TRIM29 in Cutaneous Squamous Cell Carcinoma

**DOI:** 10.3389/fmed.2021.804166

**Published:** 2021-12-20

**Authors:** Che-Yuan Hsu, Teruki Yanagi, Hideyuki Ujiie

**Affiliations:** Department of Dermatology, Faculty of Medicine and Graduate School of Medicine, Hokkaido University, Sapporo, Japan

**Keywords:** TRIM29, ATDC, squamous cell carcinoma, cell migration, keratin, FAM83H

## Abstract

Tripartite motif (TRIM) proteins play important roles in a wide range of cell physiological processes, such as signal transduction, transcriptional regulation, innate immunity, and programmed cell death. TRIM29 protein, encoded by the *ATDC* gene, belongs to the RING-less group of TRIM protein family members. It consists of four zinc finger motifs in a B-box domain and a coiled-coil domain, and makes use of the B-box domain as E3 ubiquitin ligase in place of the RING. TRIM29 was found to be involved in the formation of homodimers and heterodimers in relation to DNA binding; additional studies have also demonstrated its role in carcinogenesis, DNA damage signaling, and the suppression of radiosensitivity. Recently, we reported that TRIM29 interacts with keratins and FAM83H to regulate keratin distribution. Further, in cutaneous SCC, the expression of TRIM29 is silenced by DNA methylation, leading to the loss of TRIM29 and promotion of keratinocyte migration. This paper reviews the role of TRIM family proteins in malignant tumors, especially the role of TRIM29 in cutaneous SCC.

## Introduction

Tripartite motif (TRIM) proteins play important roles in signal transduction, transcriptional regulation, innate immunity, programmed cell death, and other cell physiological processes (1). TRIM protein dysfunction contributes to various morbid states, including viral disease, malignancy, cardiovascular disease, and neuropsychiatric disorders (2–4). More than 80 TRIM protein genes have been reported in humans. The majority of the N-terminal alignment in TRIM proteins is comprised of a RING-finger domain, one or two B boxes (the zinc-binding motif), and a coiled-coil region (5–7). The TRIM family proteins are composed of E3 ubiquitin ligase activity among the RING-finger domain. With that said, not all TRIM proteins present with a RING-finger domain; some of them are RING-less. TRIM family proteins can be subclassified into groups I to XI due to the high variability in their C-terminal functional domains, whereas the SPRY domain is predominantly conserved (6, 8). Newly discovered evidence has revealed that TRIM family proteins play a fundamental role in oncogenesis (9–11). Intriguingly, TRIM family proteins can promote either oncogenesis or tumor-suppression depending on the cancer-specific TRIM proteins. This article provides a basic overview of TRIM family proteins and discusses about their role in malignancies.

## Trim Family Proteins and TRIM29

A growing number of reports have revealed that the up- or down-regulation of TRIM proteins can promote or hinder tumorigenesis (7, 12–14). Depending on the target of the specific TRIM protein, increased expression of certain TRIM family members (e.g., TRIM25, TRIM27, and TRIM29) in tumor tissue promotes oncogenesis, and this has been correlated with poor clinical outcomes. In contrast, other TRIM proteins (e.g., TRIM3, TRIM29, and TRIM40) were found to have a tumor-suppressive effect. This dichotomic phenomenon is simultaneously observed for specific TRIM proteins (e.g., TRIM29) in different cancers.

**Figure 1:**
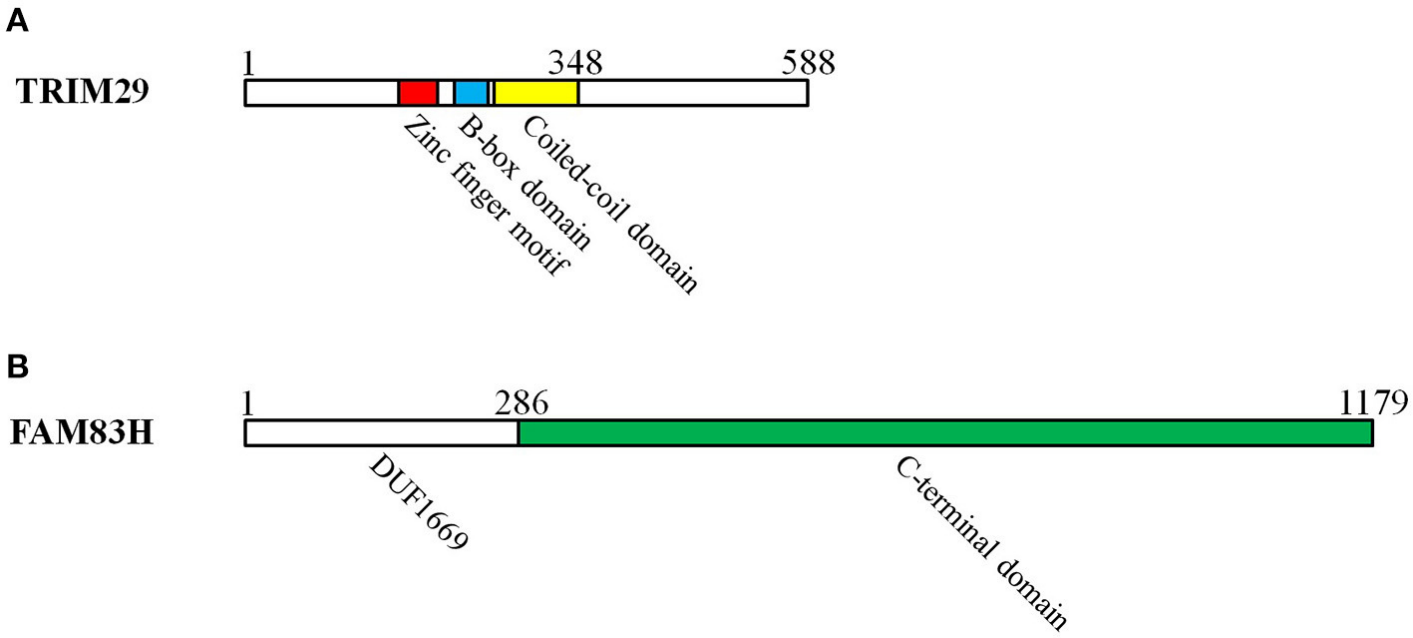
The domains of TRIM29 and FAM83H interact with keratin in cutaneous squamous cell carcinoma. **(A)** TRIM29 is pertinent to RING-less group of TRIM family proteins, which consist of four zinc finger motifs in the B-box domain and a coiled-coil domain. The zinc finger, B-box, coiled-coil, and C-terminal domains of TRIM29 are all essential for the construction of the TRIM29–keratin–FAM83H complex. **(B)** FAM83 members have a common N-terminal domain (domain unknown function 1669: DUF1669) and a C-terminal domain. The C-terminal domain of FAM83H is required for the formation of the TRIM29–keratin–FAM83H complex.

Ataxia telangiectasia is an uncommon autosomal recessive inherited disorder that affects the nervous system, the immune response, DNA repair, and the risk of cancer (15). *The ataxia-telangiectasia group D complementing (ATDC)* gene resides on human chromosome 11q23 and is involved in ataxia telangiectasia. TRIM29 protein, the product of the *ATDC* gene, belongs to the RING-less group of TRIM family proteins. It consists of four zinc finger motifs in a B-box domain and a coiled-coil domain (Figure 1A), and it makes use of the B-box domain as E3 ubiquitin ligase in place of the RING (16, 17). TRIM29 was found to be involved in the formation of homodimers or heterodimers in relation to DNA binding; additional studies have also demonstrated its role in carcinogenesis, DNA damage signaling, and the suppression of radiosensitivity (18).

### TRIM29 Protein and Innate Immunity

Innate immunity is crucial in protecting the human respiratory tract from infection. TRIM29 downregulates macrophage activation against the invasion of bacteria and viruses in the respiratory system. TRIM29 directly interacts with NEMO/IKBKG in lysosomes and induces its subsequent ubiquitination and proteolytic degradation. The expressions of downstream IFN-1 and proinflammatory cytokines are then suppressed (19). Similarly, persistent infection by a DNA virus (e.g., EBV and HSV-1) may be caused by TRIM29-induced STING1 degradation in the cGAS/STING/TBK1/IRF3 signaling pathway (20, 21). TRIM29 binds to the mitochondrial antiviral signaling protein (MAVS) and provokes its ubiquitination and degradation, leading to inhibition of the host innate defense mechanism in RNA viruses (22). In CMV-induced NK cells, TRIM29 promotes the decay of TGF-β activated kinase 1 binding protein 2 (TAB2) to decrease IFN-γ production by NK cells (23). Taken together, these studies indicate that TRIM29 is targeted by bacteria and viruses for chronic infection and immune evasion.

### TRIM29 Negatively Modulates p53

TRIM29 plays an important role in cell cycle regulation, mitosis, DNA repair, and radioresistance. Three main pathways have been identified where TRIM29 contributes to cancer formation. First, histone deacetylase 9 (HDAC9) helps to remove the acetyl group from TRIM29, decreasing the ability of TRIM29 to bind to p53, which results in the increased expression of p53-regulated genes and finally the repression of cell proliferation (24). Once TRIM29 interacts with p53, the cytoplasmic sequestration of p53 occurs and its translocation to the nucleus decreases (25). Second, TRIM29 binds to Tat-interactive protein 60 (TIP60), enhancing its breakdown, and prevents the acetylation of p53 by TIP60. The reduction of acetylated p53 causes cell growth and transformation. Lastly, the overexpression of TRIM29 in HCT116 cell lines suppresses apoptosis elicited by ultraviolet exposure (26). TRIM29 serves as a scaffold protein for recruiting DNA repair proteins into chromatin for DNA damage repair in response to ionizing radiation (27). Therefore, TRIM29 is thought to be a tumorigenic factor that negatively modulates p53, induces cell transformation, increases radiation resistance, and mounts an anti-apoptotic response.

## TRIM29 Protein in Malignancies

**Figure 2:**
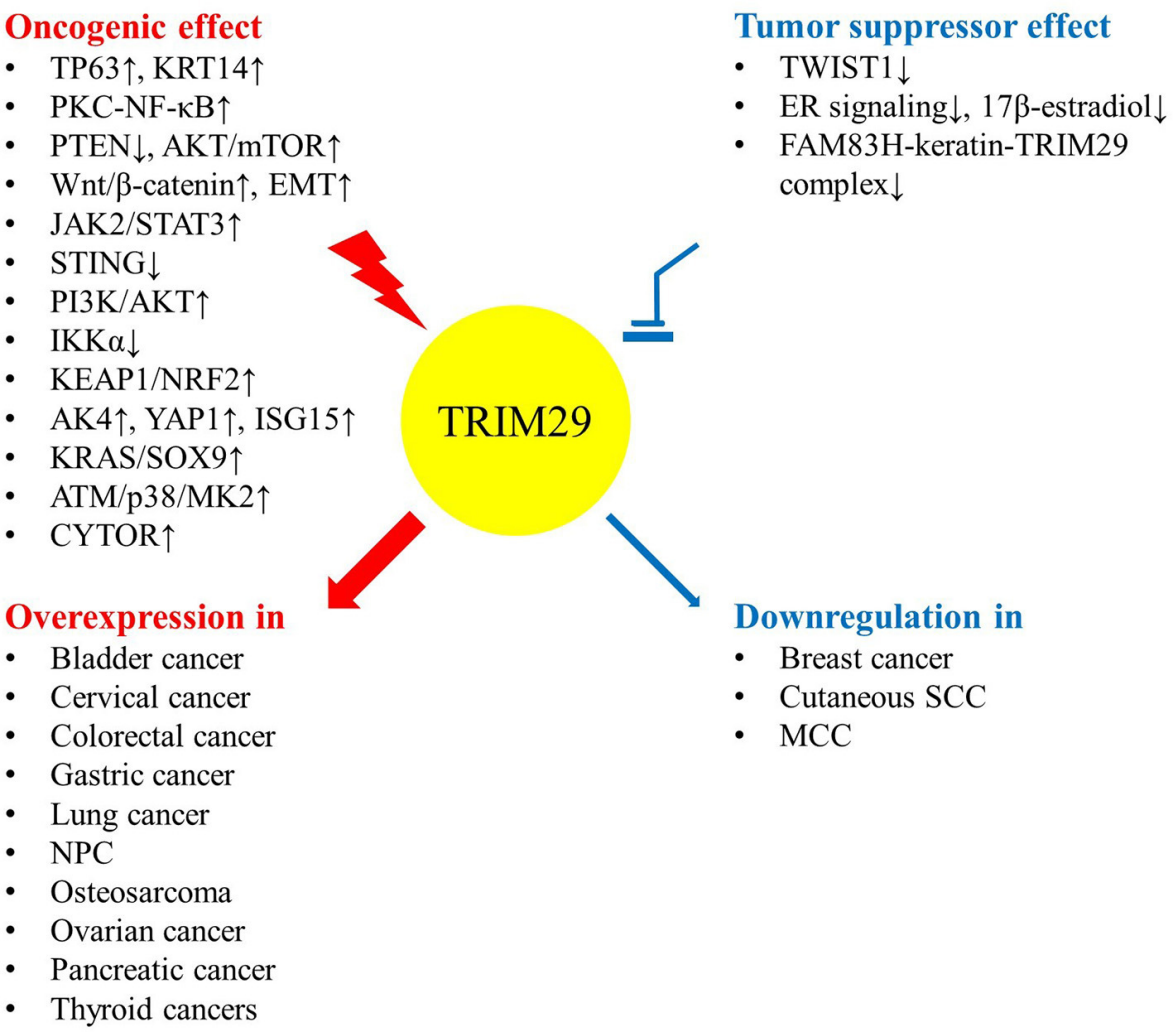
TRIM29 can act as either an oncogene or a tumor suppressor depending on the tumor type.

The expression level and function of TRIM29 contribute to human malignancies in various ways (Table 1). Even though the dysregulation of TRIM29 protein in cancer has not been fully elucidated, there are some common conditions that provide evidence for the multifaceted roles of TRIM protein (10). Elevated TRIM29 expression is accompanied by more invasive phenotypes of malignancies, including bladder, cervical, colorectal, gastric, lung, ovarian, pancreatic, and thyroid cancers; osteosarcoma; and nasopharyngeal carcinoma (NPC). Conversely, TRIM29 suppresses cell expansion, migration, and invasion in breast cancer and Merkel cell carcinoma (MCC). The low expression of this gene is associated with poor prognosis. This evidence potentially supports the dual effect of TRIM29, which can act as either an oncogene or a tumor suppressor gene, depending on the tumor type (Figure 2).

**Table 1:** Effect and expression of TRIM29 proteins in different cancers.

**Cancer type**	**Effect, Pathway, Signaling**	**TRIM29 level**	**Function**	**References**
Bladder cancer	TP63↑, KRT14↑	High	Oncogenic	(28)
	PKC-NF-κB↑	High	Oncogenic	(29)
	miR-29↓, PTEN↓	High	Oncogenic	(30)
	n.d.	High	Tumor marker	(31, 32)
Breast cancer	miR-761↑	Low	Tumor suppressor	(33)
	TWIST1↓	Low	Tumor suppressor	(34, 35)
	n.d.	Hypermethylated	Tumor suppressor	(36)
	ER signaling↓, 17β-estradiol↓	Low	Tumor suppressor	(37)
Cervical cancer	Wnt/β-catenin↑	High	Oncogenic	(38)
	TP63↑, TAp63α↑	High	Oncogenic	(39)
Colon cancer	PKM1 degradation↑	High	Oncogenic	(40)
	Wnt/β-catenin↑, CD44↑, EMT↑	High	Oncogenic	(41)
	JAK2/STAT3 signaling↑	High	Oncogenic	(42)
	n.d.	High	Tumor marker	(43)
Gastric cancer	n.d.	High	Oncogenic	(44)
	n.d.	High	Tumor marker	(45, 46)
	miR-185↓, Wnt/β-catenin↑	High	Oncogenic	(47)
HCC	Wnt/β-catenin↑	Low	Tumor suppressor	(48)
	miR-424-5p↓	High	Oncogenic	(49)
MCC	n.d.	Low in MCV^−^ MCC	Tumor suppressor	(50)
Melanoma	n.d.	High in primary, Low in metastatic	Tumor marker	(51)
NPC	Gal-9↑, STING↓	n.d.	Oncogenic	(52)
	miR-122↓, PI3K/AKT signaling↑	High	Oncogenic	(53)
	n.d.	High	Chemoresistance Tumor marker	(54, 55)
	PTEN/AKT/mTOR signaling↑	High	Oncogene	(56)
Lung	TP73-aS1↑ microRNA-34a-5p↓	High in NSCLC	Oncogenic	(57)
	E-cadherin autophagy degradation↑	High in SCC	Oncogenic	(58)
	n.d.	High in SCLC Low in NSCLC?	Therapeutic target	(59)
	n.d.	High	Oncogenic Chemoresistance	(60)
	n.d.	High	Tumor marker	(61, 62)
	IKKα↓	High in SCC	Tumor marker	(63)
	β-catenin↑	High in SCC	Tumor marker	(64)
Osteosarcoma	n.d.	High	Oncogenic	(65)
Ovarian cancer	n.d.	High	Oncogenic Chemoresistance	(66)
Pancreatic cancer	TRIM29 bind to KEAP1, NRF2↑	High	Oncogenic Chemoresistance	(67)
	TRIM29/miR-2355-3p/DDX3X/AK4↑	High	Oncogenic	(68)
	YAP1↑	High	Oncogenic	(69)
	ISG15↑	High	Oncogenic	(70)
	KRAS/TRIM29/β-catenin and SOX9↑	High	Oncogenic	(71)
	β-catenin↑, CD44↑, EMT↑ KRAS induced TRIM29↑	High	Oncogenic	(72)
	ATM/p38/MK2 pathway↑	High	Radioresistance	(73)
	n.d.	High	Oncogenic	(74)
	Wnt/β-catenin /TCF Signaling↑	High	Oncogenic	(75)
	n.d.	High	Tumor marker	(76)
Prostate cancer	n.d.	Expressed in normal cells	Tumor marker	(77)
SCC	FAM83H-keratin-TRIM29 complex↓ FAM83H↓ (Head & neck)	Low	Tumor suppressor	(78)
	Non-diffuse keratin pattern (Head & neck)	Low	Tumor suppressor	(79)
	n.d. (Oral cavity)	Low	Tumor suppressor	(80)
	Cyclin E↑ (esophagus)	High	Oncogenic	(81)
	n.d. (esophagus)	High	Oncogenic Tumor marker	(82)
	PKC↑(A431 cell)	n.d.	n.d.	(83)
Thyroid cancer	miR-195-5p↓	High	Oncogenic	(84)
	CYTOR↑, miR-873-5p↓	High	Oncogenic	(85)
	miR-761↓	High	Oncogenic	(86)
	PI3K/AKT signaling↑	High	Oncogenic	(87)

*↑, Activated; ↓, Suppressed; n.d., not determined*.

### Oncogenic Effect of TRIM29

Some studies have indicated that upregulation of TRIM29 is associated with an advanced stage and poor outcomes in many malignancies. In bladder cancer, the overexpression of TRIM29 suppresses miR-29 and subsequently activates DNA methyltransferase 3A (DNMT3A), resulting in DNA methylation and silencing of tumor suppressor PTEN (30–32). TRIM29 upregulates cyclin D1/E and Bcl family proteins by the PKC-NF-κB signaling pathway to assist cell proliferation as well as inhibit apoptosis (29). The invasive phenotype of bladder cancer occurs *via* the binding of TP63 to TRIM29 and keratin14 (KRT14), along with the enhancement of their expression (28). In cervical cancer cells, TRIM29 mediates the p63 pathway and activates Wnt/β-catenin signaling to promote cell proliferation, colony formation, migration, and invasion (38, 39). The expression of TRIM29 in colorectal cancer (CRC) tissue is significantly higher and correlates with tumor progression and poor prognosis (43). TRIM29 enhances the degradation of pyruvate kinase M1 (PKM1) and the reduction of PKM1/PKM2 ratio, which leads to stronger malignant behavior, enhanced PKM2-mediated aerobic glycolysis (the Warburg effect), and weakened oxidative phosphorylation (40). TRIM29 also plays a pro-tumorigenic role through JAK2/STAT3 signaling, induced epithelial to mesenchymal transition (EMT) via the Wnt/β-catenin signaling pathway, and CD44 expression in CRC (41, 42). In gastric cancer, TRIM29 functions as an oncogene to activate Wnt/β-catenin signaling, which can be silenced by miR-185 (44–47). The expression level of TRIM29 protein is particularly related to advanced malignant tumor stage and cancer drug resistance in NPC (54, 55). miR-335-5p and miR-15b-5p suppress the metastasis of NPC by targeting TRIM29 and inhibit the activity of the PI3K/AKT/mTOR signaling pathway (56); miR-122 inhibits tumor progression by downregulating TRIM29 and repressing the activity of PI3K/AKT signaling (53). Enrichment of galectin-9 (Gal-9) in the microenvironment and serum triggers the TRIM29-mediated K48-linked ubiquitination of STING and is associated with a more aggressive phenotype of NPC (52). In lung cancer, the upregulation of TRIM29 can cause oncogenic activity and chemoresistance (59–62). TRIM29 may also enhance tumor progression and metastasis by interaction with β-catenin and modifying E-cadherin autophagy degradation (58, 64). IKKα suppresses TRIM29 and p63 in an epigenetic manner to prevent lung squamous cell carcinoma (SCC) development, but TP73-AS1 serves as a molecular sponge for miR-34a-5p to prevent TRIM29 suppression (57, 63). In osteosarcoma and ovarian cancer, TRIM29 induces EMT and chemoresistance, respectively (65, 66).

TRIM29 is thought to be a molecular marker and oncogene for pancreatic ductal adenocarcinoma (74, 76). It binds to Disheveled-2 (Dvl-2) and components of the β-Catenin destruction complex to stabilize β-catenin, resulting in the activation of Wnt/β-catenin/TCF signaling pathway (75). The activation of β-catenin signaling further upregulates CD44, which induces EMT characterized by the expression of Zeb1 and Snail1 (72). TRIM29 can also directly bind to Yes-associated protein 1 (YAP1) to reduce its ubiquitination and degradation, and thus promote the proliferation of pancreatic cancer cells (69). In the presence of oncogenic KRAS, TRIM29 subsequently upregulates SRY-Box transcription factor 9 (SOX9), which accelerates the formation of pancreatic intraepithelial neoplasia and progression to invasive carcinoma (71). The DNA damage sensor ataxia telangiectasia mutated (ATM) is activated by exposure to ionizing radiation, and it causes MAPKAP kinase 2 (MK2) kinase phosphorylation in a p38 kinase-dependent process. The phosphorylated MK2 induces TRIM29 phosphorylation, resulting in a radioresistant response by pancreatic cancer cells (73). TRIM29 interacts with Kelch-like ECH-associated protein 1 (KEAP1) to prevent the degradation of nuclear factor erythroid 2-related factor 2 (NRF2), which promotes antioxidant response element and enhanced chemoresistance (67). The cancer stem cell-like features of pancreatic ductal adenocarcinoma are maintained by the autocrine stimulation of extracellular interferon-stimulated gene 15 (ISG15), which is modulated by TRIM29 via Calpain-3 (CAPN3)-mediated posttranslational degradation (70). In addition, TRIM29 controls the transcripts of adenylate kinase 4 (AK4) via posttranscriptional regulation to alter the bioenergetics in pancreatic cancer (68). In thyroid carcinoma, high expression of TRIM29 activates PI3K/AKT signaling, which circumvents cell cycle arrest at the G0/G1 phase and enhances cancer cell resistance to chemotherapy (87). LncRNA HOXA11-AS has been identified as a molecular sponge that competes with endogenous miR-761, which downregulates TRIM29 (86). Similarly, TRIM29 inhibits miR-873 biogenesis by LncRNA CYTOR sponging to upregulate FN1 and promote the progression of papillary thyroid cancer cells (85). In papillary thyroid carcinoma, LncRNA 00324 is characterized as a microRNA sponge to miR-195-5p to maintain the oncogenic effect of TRIM29 (84).

### Tumor-Suppressive Effect of TRIM29

TRIM29 can antagonize 17β-estradiol-induced cell proliferation, interrupt the induction of estrogen-responsive genes, and block the upregulation of the TWIST1 oncogene in response to hypoxic stress (34, 35, 37). However, TRIM29 is underexpressed in primary breast tumors. It is silenced by gene hypermethylation or by a specific microRNA, miR-761, leading to the aggressive behavior of breast cancer (33, 36). In Merkel cell polyomavirus-uninfected (MCV^−^) MCC, more aggressive disease may be linked to the loss of TRIM29 expression (50). More studies are warranted to verify these early findings.

### Equivocal or Unknown Status of TRIM29 in Specific Malignancy

The function of TRIM29 in hepatocellular carcinoma (HCC) is ambiguous, and it has been reported that the silencing of TRIM29 is associated with cell proliferation, tumor formation, cell migration, and cell invasion. TRIM29 possesses a tumor-suppressor feature via inhibition of the Wnt/β-catenin signaling pathway (48). However, another study indicated that miR-424-5p, a tumor-suppressor miRNA, targets the TRIM29 gene and reverses its oncogenic effects (49). Therefore, more evidence is required to elucidate the exact role of TRIM29 in HCC. Interestingly, TRIM29 expression was found to be lower in metastatic melanoma but higher in primary melanoma. The status of TRIM29 in melanoma also needs further confirmation (51). In prostate cancer, TRIM29 is expressed in normal cells but not in cancer cells (77).

## TRIM29 Protein and SCC

Cutaneous squamous cell carcinoma (cSCC), the second most common non-melanoma skin malignancy, contributes to more than 20% of skin cancers in Caucasian populations (88). cSCC derives from the malignant transformation of epithelial keratinocytes, and cumulative UV damage in the sun-exposed epithelium is the major cause of cSCC (89, 90). Other risks inclined toward morbidity include male gender, elderly age, Fitzpatrick skin type I to III, immunocompromise, hematologic cancer, HPV infection, chronic wounds, scars, several hereditary syndromes (e.g., epidermolysis bullosa, oculocutaneous albinism, xeroderma pigmentosum), and environmental exposure to arsenic or coal tar. Regarding the genetic changes, cSCC is the second most mutated skin cancer after basal cell carcinoma. The most frequent mutation in cSCC is the tumor suppressor gene *p53*. UV-induced *p53* mutation prevents keratinocytes from apoptosis and promotes the precancerous clonal expansion of abnormal cells. The two other common tumor suppressor genes (*CDKN2A* and *NOTCH*), as well as the oncogenic *RAS* gene, all participate in the initial event of oncogenesis. *Epidermal growth factor receptor (EGFR)* gene amplification along with protein overexpression is highly correlated with aggressive phenotypes and poor outcomes (91). As a consequence of the accumulation of these altered alleles, the skin in the affected area evolves from hyperplasia and dysplasia to invasive carcinoma.

An early study showed that the quantity of TRIM29 is markedly higher in epidermoid cells (cSCC cell line A431) than in fibroblast cell lines. TRIM29 is the substrate of serine/threonine protein kinase C (PKC), and it may regulate the cell response to ionizing radiation via the PKC signaling pathway, but the role of TRIM29 in tumorigenesis has not been determined (83). Furthermore, elevated TRIM29 expression is associated with tumor growth and proliferation via the regulation of cyclin E in esophageal SCC patients. TRIM29 might be a useful marker for evaluating prognosis (81, 82). The oncogenic effect of TRIM29 is also found in lung SCC. Conversely, new evidence points to the tumor-suppressive effect of TRIM29 in cSCC (78–80). This may be due to tissue-specific gene regulation differences between internal organs and keratinocytes.

### Expression of TRIM29 and FAM83H in CSCC

The expression of TRIM29 is decreased in cutaneous head and neck SCC but is normal in healthy skin and benign tumors. Unusual hypermethylation in the CpG island of the TRIM29 promoter suppresses the expression of TRIM29. Decreased TRIM29 is related to more aggressive clinical behavior (79). Such results strongly suggest that TRIM29 is a useful diagnostic and prognostic biomarker for cSCC. Furthermore, the subcellular localization of TRIM29 is mainly found in the cytoplasm, consistent with TRIM29 acting as a tumor suppressor in breast and prostate cancers. The level of TRIM29 in the cytosol alters the distribution pattern of keratin in cSCC, with low expression showing a perinuclear pattern and high expression presenting a diffuse pattern. Another experiment confirmed that keratin-interacting protein FAM83H (Family With Sequence Similarity 83 Member H) could be a new interactor with TRIM29 (Figure 1B) (78). FAM83H expression is also lower in cSCC than in normal skin, and the level shows an association with different cancer stages. FAM83H knockdown increase the solubility of keratin 5 and 14 in the soluble fraction of cancer cell lysates. The change in solubility of keratin could assist in the redistribution of keratins during cell division (92). This would mean that the loss of FAM83H induces the reorganization of keratin and promotes SCC cell migration, invasion, and metastasis *in vivo*. Additionally, FAM83H knockdown enhances the level of integrin β4 at the wound edge, which reinforce keratinocytes migration close to that edge (93). This means that FAM83H may also be an essential molecule in the cell motility and oncogenesis of cSCC.

### The TRIM29–Keratin–FAM83H Complex Regulates Keratin Distribution

**Figure 3:**
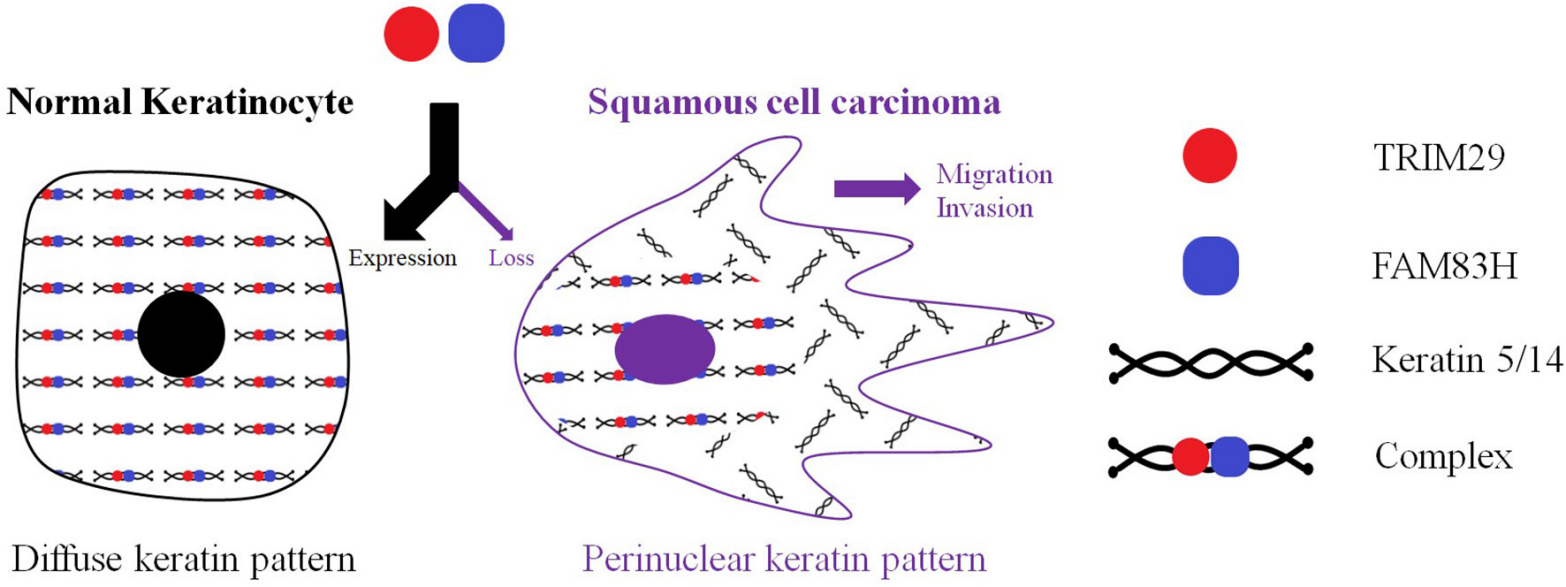
TRIM29 was proven to colocalize with keratin 5, keratin 14, and FAM83H in the cytosol, and to form the TRIM29–keratin–FAM83H complex. FAM83H and TRIM29 can be characterized as sharing similar features in regulating keratin distribution and cell migration. The TRIM29–keratin–FAM83H complex is thought to regulate the keratin distribution in cutaneous SCC.

Keratin filaments are of fundamental importance in protecting epithelial cells from damage or stress. Some current studies provide supporting evidence that keratin plays a role in the regulation of cell polarization, migration, and signal transduction (94). The distribution of intracellular keratin is involved in cancer invasion, metastasis, and treatment response. In cSCC, TRIM29 has been proven to colocalize with keratin 5, keratin 14, and FAM83H in the cytosol, and to form the TRIM29–keratin–FAM83H complex. The zinc finger, B-box, coiled-coil, and C-terminal domains of TRIM29 and the C-terminal domain of FAM83H are all essential for the construction of the TRIM29–keratin–FAM83H complex (Figure 1), and FAM83H or TRIM29 binds to keratins separately (78, 79). FAM83H and TRIM29 can be characterized as sharing similar features in the regulation of keratin distribution and cell migration. Consequently, the TRIM29–keratin–FAM83H complex is thought to regulate keratin distribution in cSCC (Figure 3).

## Conclusion

We have summarized recent advances in our understanding of the multiple roles of TRIM29 in innate immunity and oncogenesis. In the past few years, more studies have confirmed that TRIM29 has “two-faced” effects in various malignancies. With reference to the finding that low TRIM29 expression is closely associated with poor prognosis in cSCC, it stands for promising therapeutic targets and modern tumor markers for early identification and risk evaluation. The modulation of the TRIM29–keratin–FAM83H complex could be an interventional strategy, via DNA and RNA, as well as epigenetic therapy. Also, injury to the epidermis provokes the keratin filaments to redistribute from a diffuse cytoplasmic pattern to a perinuclear distribution and prompts the upregulation of integrin β4, which cause keratinocytes to migrate to the wound. The TRIM29–keratin–FAM83H complex seems to be involved in wound healing. This raises the novel idea that developing a TRIM29 inhibitor could contribute to wound healing by disturbing the formation of this complex. We expect these concepts to be gradually put to practical use.

## Author Contributions

C-YH: designed this project, acquired the data and references, and wrote the manuscript. TY: designed this project and wrote the manuscript. HU: supervised this project, analyzed the data, and wrote the manuscript. All authors contributed sufficiently and met the criteria for authorship.

## Conflict of Interest

The authors declare that the research was conducted in the absence of any commercial or financial relationships that could be construed as a potential conflict of interest.

## Publisher's Note

All claims expressed in this article are solely those of the authors and do not necessarily represent those of their affiliated organizations, or those of the publisher, the editors and the reviewers. Any product that may be evaluated in this article, or claim that may be made by its manufacturer, is not guaranteed or endorsed by the publisher.
